# Efficient enzyme-free method to assess the development and maturation of the innate and adaptive immune systems in the mouse colon

**DOI:** 10.1038/s41598-024-61834-5

**Published:** 2024-05-14

**Authors:** Nejia Lassoued, Alexis Yero, Mohammad-Ali Jenabian, Rodolphe Soret, Nicolas Pilon

**Affiliations:** 1 Molecular Genetics of Development Laboratory, Department of Biological Sciences, Université du Québec à Montréal, Montreal, QC Canada; 2 Centre d’excellence en recherche sur les maladies orphelines – Fondation Courtois (CERMO-FC), Université du Québec à Montréal, Montreal, QC Canada; 3 Human Immuno-Virology Laboratory, Department of Biological Sciences, Université du Québec à Montréal, Montreal, QC Canada; 4 Department of Pediatrics, Université de Montréal, Montreal, QC Canada

**Keywords:** Lymphoid cells, Myeloid cells, Multi-color flow cytometry, Mechanical dissociation, Mouse colon, Immune cell isolation, Colon

## Abstract

Researchers who aim to globally analyze the gastrointestinal immune system via flow cytometry have many protocol options to choose from, with specifics generally tied to gut wall layers of interest. To get a clearer idea of the approach we should use on full-thickness colon samples from mice, we first undertook a systematic comparison of three tissue dissociation techniques: two based on enzymatic cocktails and the other one based on manual crushing. Using flow cytometry panels of general markers of lymphoid and myeloid cells, we found that the presence of cell-surface markers and relative cell population frequencies were more stable with the mechanical method. Both enzymatic approaches were associated with a marked decrease of several cell-surface markers. Using mechanical dissociation, we then developed two minimally overlapping panels, consisting of a total of 26 antibodies, for serial profiling of lymphoid and myeloid lineages from the mouse colon in greater detail. Here, we highlight how we accurately delineate these populations by manual gating, as well as the reproducibility of our panels on mouse spleen and whole blood. As a proof-of-principle of the usefulness of our general approach, we also report segment- and life stage-specific patterns of immune cell profiles in the colon. Overall, our data indicate that mechanical dissociation is more suitable and efficient than enzymatic methods for recovering immune cells from all colon layers at once. Additionally, our panels will provide researchers with a relatively simple tool for detailed immune cell profiling in the murine gastrointestinal tract, regardless of life stage or experimental conditions.

## Introduction

The immune system plays a critical role in maintaining homeostasis and protecting the body against harmful pathogens. Within the intricate network of immune defenses, the gastrointestinal tract houses a complex and dynamic immune system that serves as the first line of defense against a myriad of microorganisms encountered through food and environmental exposure^1–6^. The gut immune system is tasked with the challenging dual role of tolerating and interacting with the vast community of commensal microorganisms residing in the intestine while swiftly responding to potential threats posed by invading pathogens^7–9^. This delicate balance is essential for preserving gut health and overall immune function.

The immune system begins its development during embryogenesis, playing a crucial role in protecting the developing fetus from neonatal infections^10–12^. Initially, the fetal immune system primarily consists of primitive immune cells, including immature T and B lymphocytes, natural killer cells, monocytes, macrophages and dendritic cells^13–17^. As embryonic development progresses, the immune system undergoes diversification and specialization, enabling it to respond to encountered antigens^10,18^. Yet, a pivotal milestone in the development of the gut immune system occurs at birth. During vaginal delivery, the newborn is exposed for the first time to a diverse range of commensal bacteria while passing through the vaginal canal^19,20^. These commensal bacteria colonize the baby’s digestive tract and thereby play a fundamental role in the post-natal maturation of the developing gastrointestinal tract, including the immune system^21–25^. Notably, some of these bacteria actively contribute to the production of short-chain fatty acids, such as butyrate, which serves as an energy source for mucosal cells while also helping to strengthen epithelial junctions, balance nitrergic and cholinergic neuron subtypes in the enteric nervous system, and regulate immune responses^26–31^. The immune system then continuously matures as the external environment continuously exposes the newborn to various antigens^1,21,32,33^. In adulthood, the mature immune system within the gut comprises approximately 70% of the total immune cell populations^34–37^.

Multi-parameter flow cytometry is ideally suited for quantitative and qualitative analyses of individual immune cell populations. This technique enables the comprehensive characterization of diverse immune cells from the gut wall, including their phenotypes, activation status, functional attributes, and distribution patterns. Although the intestinal mucosa/submucosa have been the primary focus of most of these studies due to the abundance of immune cells in these tissue layers^5,38–42^, the muscle layers have nonetheless been the center of interest of others^43–47^. Ideally, profiling immune cells from all layers at once would be needed for a true comprehensive understanding of immune cell profiles. However, such analysis is complicated by the different structural properties of each layer (e.g., soft mucosa *vs* firm muscle), implying that a tissue dissociation method that works well for one layer might not work well for another. Consequently, a wide range of methods based on enzymatic and/or mechanical processing have been developed for dissociating gastrointestinal tissues^38,48–50^. Notably, several cocktails of enzymes that include different types/concentrations of collagenase, liberase and/or dispase have been previously described, with the consequence that a standardized approach remains elusive^33,38,40,41,48,51–53^.

The main objective of the current study was to develop a standardized-like and relatively simple method for the global profiling of lymphoid and myeloid cell lineages from the whole mouse colon. To this end, we first compared the efficacy of three tissue dissociation methods (two enzymatic and one mechanical) based on total cell yield, viability, and preservation of cell-surface markers. We then addressed another gap in the field in that most studies predominantly focus on either lymphoid or myeloid cell populations, rarely both, and with a limited number of markers^54–56^. Here, we describe two antibody panels targeting a total of 26 markers that enable concomitant characterization of both innate and adaptive immune cells. Combining mechanical tissue dissociation with these antibody panels allowed us to evaluate a broad range of lymphoid and myeloid cells from all layers of the mouse colon at once. Importantly, the procedure described herein is relatively simple and expeditious, also offering the flexibility to modify cell markers based on the research questions of interest.

## Methods

### Animals

FVB mice initially purchased from Charles River (strain code 207) were maintained and bred in individually ventilated cages within the conventional animal facility of the Université du Québec à Montréal, under 12 h light–12 h dark cycles (7AM to 7PM) and with ad libitum access to regular chow diet (Charles River Rodent Diet #5075, Cargill Animal Nutrition). Organs and blood samples were collected from pre-weaned juvenile (postnatal day [P] 20) and weaned adult (P120) animals. Prior to anesthesia, mice were weighed and sexed based on external genitalia. Both sexes were included in the current study (n = 2 males and 1 female per experiment). Whole blood (200 µl from P20 mice and 400 µl from P120 mice) was collected via intracardiac puncture of mice kept under deep anesthesia using isoflurane. Subsequently, mice were euthanized using CO2 and transferred to the laboratory for colon and spleen collection. All experimental procedures involving mice were approved by the institutional review board and ethics committee of the Université du Québec à Montréal (CIPA protocol #959) following the biomedical research guidelines of the Canadian Council of Animal Care (CCAC). All data obtained with mice are reported in accordance with the ARRIVE guidelines 2.0^57^.

### Preparation of single-cell suspensions

Dissected samples of whole spleen and colon were immediately placed in a sterile Petri dish containing cold RPMI 1640 1 × medium (WISENT INC, #350-000-CL) on ice. For spleen samples, single-cell suspensions were directly obtained via gentle manual grinding with the flat head of a syringe plunger on a 70 µm cell strainer (Movie S1). For colon samples, mesenteric fat was removed, and the colon was divided into two regions: proximal colon and distal colon. Each region was opened longitudinally along the mesenteric border, flushed with RPMI medium, and processed for tissue dissociation. To test the three different dissociation methods, each region was further sub-divided into three fragments of equal length. Each of the three dissociation methods was tested using a full-thickness fragment (i.e., including all unaltered tissue layers) from the proximal colon combined to a full-thickness fragment from the distal colon. Mechanical dissociation of these colon samples was performed as described above for spleen samples, but with a slightly stronger manual pressure on tissue, placing mucosal side on the cell strainer (Movie S2). Enzymatic dissociation was performed using a previously published method^48,52,53^. Two different enzymatic cocktails were tested, both prepared in RPMI medium supplemented with DNase I (0.1 mg/ml, Sigma-Aldrich #DN25): one cocktail contained Dispase II (1.3 mg/ml, Life Technologies #17105-041) and Collagenase I (0.4 mg/ml, Sigma-Aldrich #C2674), while the other contained Liberase TL (0.15 mg/ml, Roche #05401020001) and Collagenase V (0.4 mg/ml, Sigma-Aldrich #C9263). To compare efficiency of the mechanical approach on full-thickness colon *vs* mucosal/submucosal layers only, proximal and distal colon regions were further sub-divided into two fragments of equal length, with one of these further processed to peel off the mucosal/submucosal layers from the muscle/serosa layers, as previously described^58–60^. To this end, full-thickness samples were immobilized with mucosa side up by pinning onto SYLGARD-coated (Thermo Fisher Scientific, #50822180) Petri dishes and were subsequently carefully stripped under a dissecting microscope to separate the soft mucosal and associated submucosal layers from the firm muscle/serosal layers. After extensive washing in RPMI, corresponding fragments (full-thickness or mucosa/submucosa-only) from the proximal colon and distal colon were then combined for analysis.

Each resulting single-cell suspension was filtered through a 40 µm cell strainer and incubated in RPMI medium supplemented with 10% FBS for 2 h at 37 °C to promote the re-appearance of cell surface markers. After gentle centrifugation (1500 RPM during 5 min) and resuspension in 1 ml of RPMI + 2% FBS, viable cells were counted using a hemocytometer with trypan blue counterstain. For each sample, two tubes containing between 600,000 and 1,000,000 live cells in 200 µl of RPMI were prepared and processed in parallel for staining with antibody panels for lymphoid and myeloid cell lineages as described below. Additional tubes containing either non-stained cells or cells stained with viability markers only (see Tables 1, 2) were also prepared for compensation prior to data acquisition with the flow cytometer.

**Table 1:** Antibodies used for lymphoid cell phenotyping in mice.

Marker	Fluorochrome	mAb clone	Purpose	Source	Cat. #
Aqua Vivid	Vivid 405 nm	–	viability	Invitrogen	L34957
CD45	BV421	30-F11	pan-leukocytes	BD Horizon	563890
CD3	PE-cy5	145-2C11	pan-T-cells	BD Pharmingen	553065
CD4	BV650	GK1.5	CD4 T-cells	BD Horizon	563232
^1^CD8	APC-R700	53–6.7	CD8 T-cells	BD Horizon	564983
^1^CD19	BV711	1D3	B-cells	BD Horizon	563157
^1^CD25	PE	PC61	Treg and activation	BD Pharmingen	553866
^2^CD8	BV711	53–6.7	CD8 T-cells	BD Horizon	563046
^2^NK1.1	PE	PK136	NK cells	BD Pharmingen	553165
^2^CD103	APC-R700	M290	tissue-resident *vs* circulating	BD Horizon	565529
CD44	FITC	IM7	naive *vs* memory	BD Pharmingen	553133
CD62L	BV605	MEL-14	naive *vs* memory	BD Horizon	563252
CD69	APC-cy7	H1.2F3	activation	BD Pharmingen	561240
CD39	Alexa Fluor 647	Duha59	effector (ectonucleotidase)	Biolegend	143808
CD73	PE-cy7	TY/11.8	effector (ectonucleotidase)	Biolegend	127224
FoxP3	PE-CF594	MF23	Treg	BD Horizon	562466
RORγt	BV786	Q31-378	Th17 cells and ILC3	BD Horizon	564723

In a variant of the panel, antibodies identified with the #1 superscript are replaced by those with the #2 superscript. Treg, regulatory T-cells; NK, natural killer; Th17, T-helper 17; ILC3, innate lymphoid cells type 3.

**Table 2:** Antibodies used for myeloid cell phenotyping in mice.

Marker	Fluorochrome	mAb clone	Purpose	Source	Cat. #
Live/Dead	APC-H7	–	viability	Invitrogen	L10119
CD45	BV421	30-F11	pan-leukocytes	BD Horizon	563890
CD11b	BV605	M1/70	Mo, MΦ and DC	BD Horizon	563015
CD11c	BV711	HL3	MΦ and DC	BD Horizon	563048
CD64	BV650	X54-5/7.1	Mo, MΦ, Mo-derived DC (Fc receptor)	BD OptiBuild	740622
CD192 (CCR2)	BV786	747966	Mo subsets (cytokine receptor)	BD OptiBuild	475301
I-A/I-E (MHC II)	Alexa Fluor 488	M5/114.15.2	MΦ and DC (antigen presenting cells)	BD Pharmingen	562352
CX3CR1	PerCP/Cy5.5	SA011F1	MΦ and DC (cytokine receptor)	Biolegend	149010
F4/80	PECF594	T45-2342	MΦ subsets	BD Horizon	565613
Ly6C	PE-cy7	AL-21	Mo *vs* MΦ	BD Pharmingen	560593
TIM-4	BV510	21H12	tissue-resident MΦ (long lived)	BD OptiBuild	742774
CD103	APC-R700	M290	tissue resident *vs* circulating DC	BD Horizon	565529
CD115	APC	AFS98	Mo and MΦ (CSF receptor)	Biolegend	135510
CD163	PE	TNKUPJ	MΦ subsets	eBioscience	12-1631-82

Mo, monocytes, MΦ, macrophages, DC, dendritic cells.

### Staining of single-cell suspensions

Single-cell suspensions from colon, spleen, and whole blood samples were stained with cocktails of fluorescently labeled antibodies designed to mark lymphoid and myeloid cell populations as described in Tables 1 and 2, respectively. For colon and spleen samples, cells were first stained for extracellular markers during 1 h at 4 °C. These samples were then washed with 1 ml of PBS, centrifuged (1500 RPM during 5 min) and the medium was partially removed to leave cells in 200 μl. For the myeloid panel, cells were fixed using CytoFix (BD CytoFix™, #554655) according to the manufacturer’s instructions. For the lymphoid panel, cells were instead fixed and permeabilized using the Transcription Factor Buffer Set (BD Pharmingen™, #562574) according to the manufacturer’s instructions, and then processed for additional intracellular staining with FoxP3 and ROR$\upgamma$t antibodies (Table 1) during 1 h at 4 °C. After completion of the fixation and/or additional intracellular staining steps for both panels, cells were washed and centrifuged to leave them in 200 μl as described above. All tubes were finally kept at 4 °C protected from light until data acquisition in the flow cytometer. For blood samples, the same protocol as described above was used with an additional post-staining step to lyse red blood cells with relevant lysis buffer (BD Pharm Lyse™, # 555899) according to the manufacturer’s instructions.

### Flow cytometry

All samples were analysed using a LSR Fortessa™ X-20 flow cytometer (BD Biosciences), with 3-laser configuration (Blue, Violet, Yellow-Green). To ensure accurate fluorescence compensation for each panel, a FACS tube was prepared for each antibody by adding 100 μl PBS, 1 drop of compensation beads (Invitrogen™, #01–2222-41), and 1 μl of the specific antibody. Successful compensation was achieved when there was no overlap between fluorochromes, also considering signals obtained for non-stained cells and cells stained for viability only. Data from each sample were finally acquired and recorded until reaching the desired number of events. Cell populations were manually gated using the FlowJo software V10.8.0 (BD Biosciences), which was also used to globally visualize data via 2-dimensional t-SNE analysis.

### Statistical analysis

All experiments were performed using three independent biological replicates, with data expressed as the mean ± standard error of the mean (SEM). The significance of differences was determined using two-tailed Student’s *t*-tests in GraphPad Prism software V9.5.1. Only statistically significant differences (*p* < 0.05) are indicated in the figures (*, **, ***, **** represent *p* < 0.05, *p* < 0.01, *p* < 0.001, *p* < 0.0001, respectively).

## Results

### Mechanical dissociation best preserves cell-surface markers in immune cells from full-thickness colon samples from P20 mice

**Figure 1:**
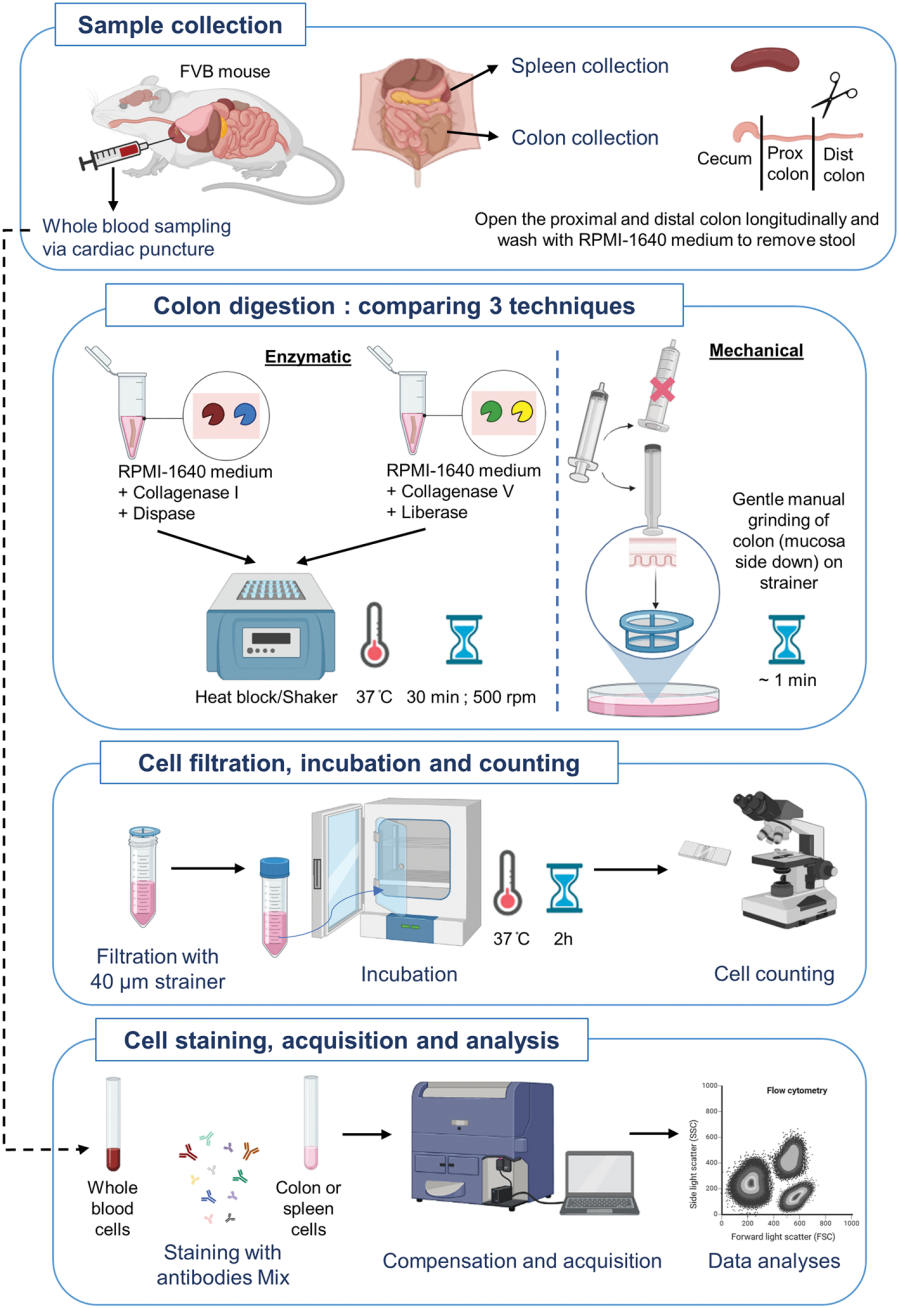
Schematic representation of immune cell isolation protocols for flow cytometry analysis. This illustration was created with BioRender.com.

**Figure 2:**
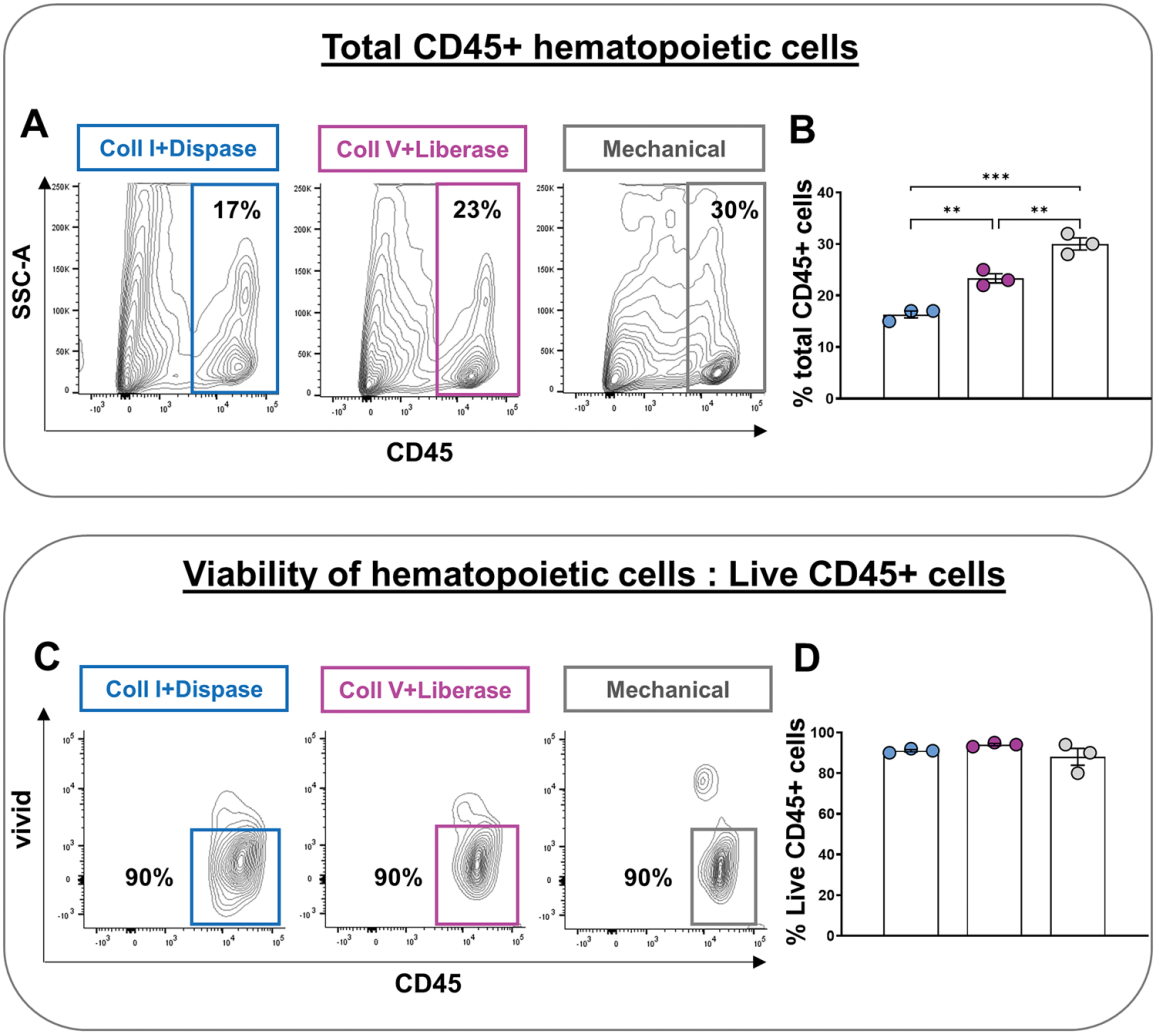
Mechanical disruption of mouse colon results in higher immune cell yields than enzymatic digestion, without impacting viability. (**A**,**B**) The mechanical technique results in increased frequencies of total CD45+ immune cells, as shown in representative contour plots (**A**) and associated quantitative analyses (**B**). (**C**,**D**) Similar percentages of live CD45+ hematopoietic cells (gated on singlets) were retrieved from the different isolation protocols, as shown in representative contour plots (**C**) and associated quantitative analyses (**D**). N = 3 biological replicates; **P ≤ 0.01 and ***P ≤ 0.001; Student’s *t*-test.

**Figure 3:**
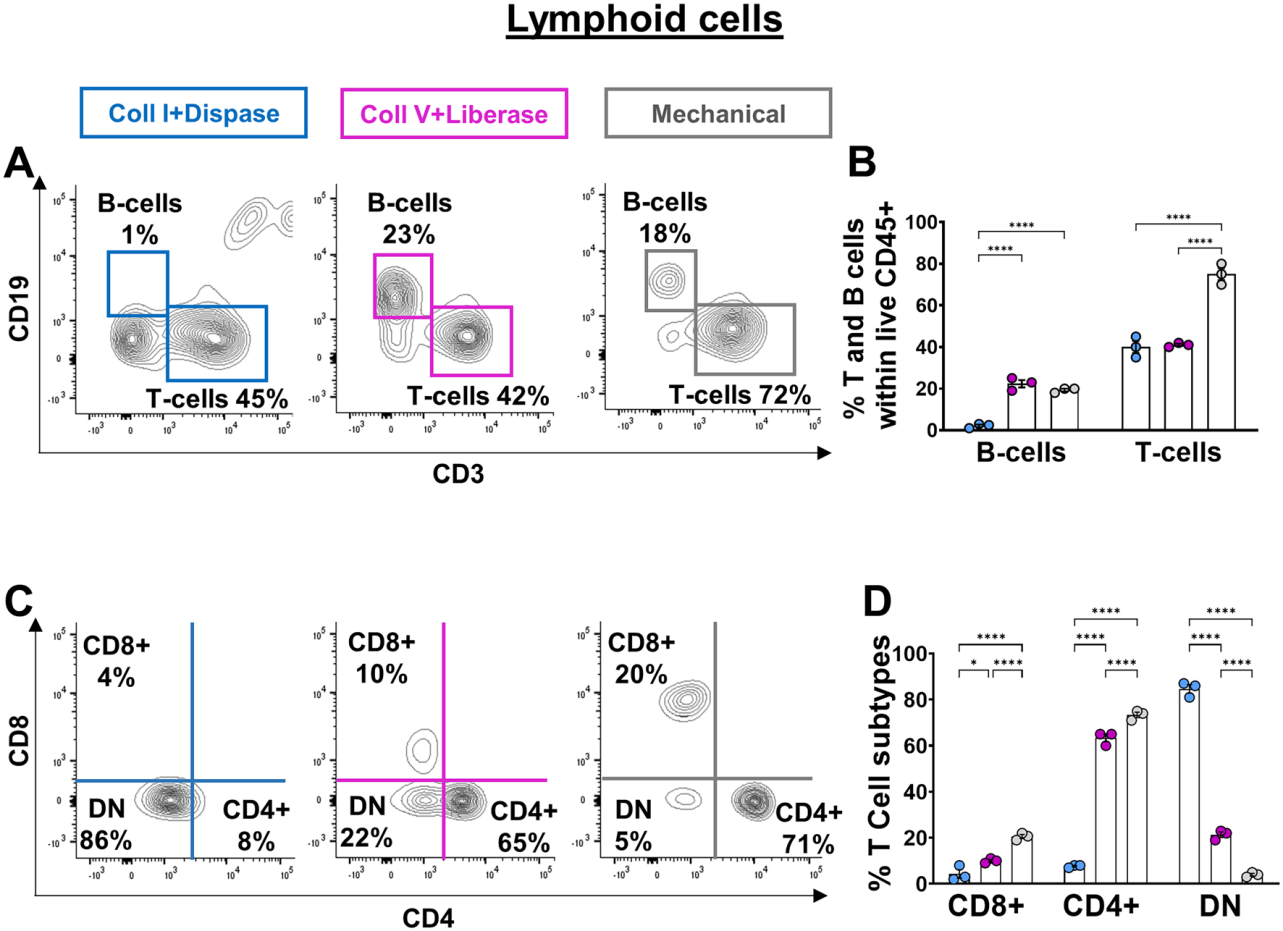
Mechanical disruption of mouse colon best preserves the cell surface markers of gut lymphoid cells. (**A**–**D**) Both enzymatic cocktails negatively affect the detection of the main populations of lymphoid cells (B-cells *vs* T-cells in panels A-B; CD4+ *vs* CD8+ T-cells in panels **C**,**D**), as shown in representative contour plots (**A**,**C**) and associated quantitative analyses (**B**,**D**). N = 3 biological replicates; *P ≤ 0.05 and ****P ≤ 0.0001; Student’s *t*-test.

**Figure 4:**
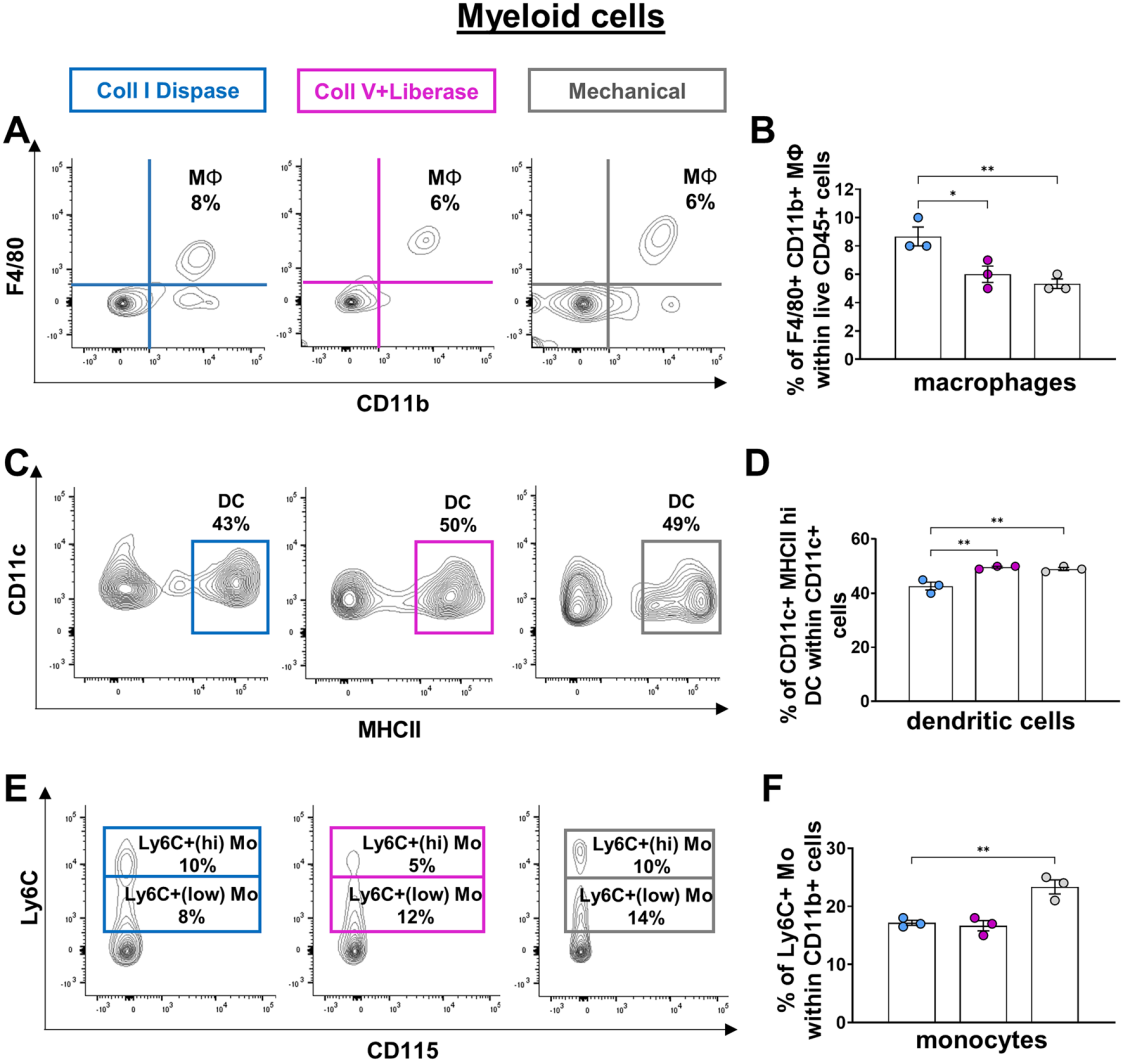
Mechanical disruption of mouse colon best preserves the cell surface markers of gut myeloid cells, except for macrophages. (**A**,**B**) The Collagenase I & Dispase II cocktail is slightly better than both the Collagenase V & Liberase LT cocktail and mechanical disruption for detecting F4/80+ CD11b+ macrophages, as shown in representative contour plots (**A**) and associated quantitative analyses (**B**). (**C**,**D**) Mechanical disruption and the Collagenase V & Liberase LT cocktail are both slightly more efficient than the Collagenase I & Dispase II cocktail for detecting CD11c+ MHCII+ (hi) dendritic cells, as shown in representative contour plots (**C**) and associated quantitative analyses (**D**). (**E**,**F**) Mechanical disruption is more effective than both enzymatic cocktails for detecting Ly6C+ monocytes, as shown in representative contour plots (**E**) and associated quantitative analyses (**F**). N = 3 biological replicates; *P ≤ 0.05 and **P ≤ 0.01; Student’s *t*-test. MΦ, macrophages; Mo, monocytes; DC, dendritic cells.

To determine the best method of colon dissociation for analyzing immune cells by flow cytometry, we tested 3 different techniques on full-thickness samples (i.e., including all unaltered tissue layers) from pre-weaned P20 mice: two enzymatic (based on either Collagenase I & Dispase II or Collagenase V & Liberase TL) and one mechanical (Fig. 1). We first analyzed the impact of these methods on the total number of recovered CD45+ hematopoietic cells, which revealed markedly greater efficiency of the mechanical approach over both enzymatic ones (Fig. 2A,B). Importantly, we also found that this gain of recovery efficiency was not made at the expense of viability, as all three methods gave similar proportions of viable CD45+ cells (Fig. 2C,D). Analysis of lymphoid and myeloid cell lineages with some general markers then revealed that the greater efficiency of the mechanical technique was in fact more specific to lymphoid cells. Frequencies of CD19+ B-cells, CD3+ T-cells, CD4+ T-cells and CD8+ T-cells were all higher with the mechanical technique (Fig. 3A–D), with an exception for the frequency of CD19+ B-cells which was equalized by the enzymatic approach based on Collagenase V & Liberase TL (Fig. 3A,B). The mechanical and the Collagenase V & Liberase TL approaches also yielded similar frequencies of F4/80+ CD11b+ macrophages (Fig. 4A,B) and CD11c+ MHCII+ dendritic cells (Fig. 4C,D), which in this case were either lower or higher than with the Collagenase I & Dispase II approach, respectively. Yet, the mechanical approach again outperformed both enzymatic techniques in the identification of Ly6C+ monocytes (Fig. 4E,F). Considering all of the above, we conclude that mechanical dissociation best balances total cell recovery yields with cell viability and presence of cell-surface markers in lymphoid and myeloid cells.

### Full-thickness colon samples enable the characterization of a diversified pool of gastrointestinal immune cells in P20 mice

**Figure 5:**
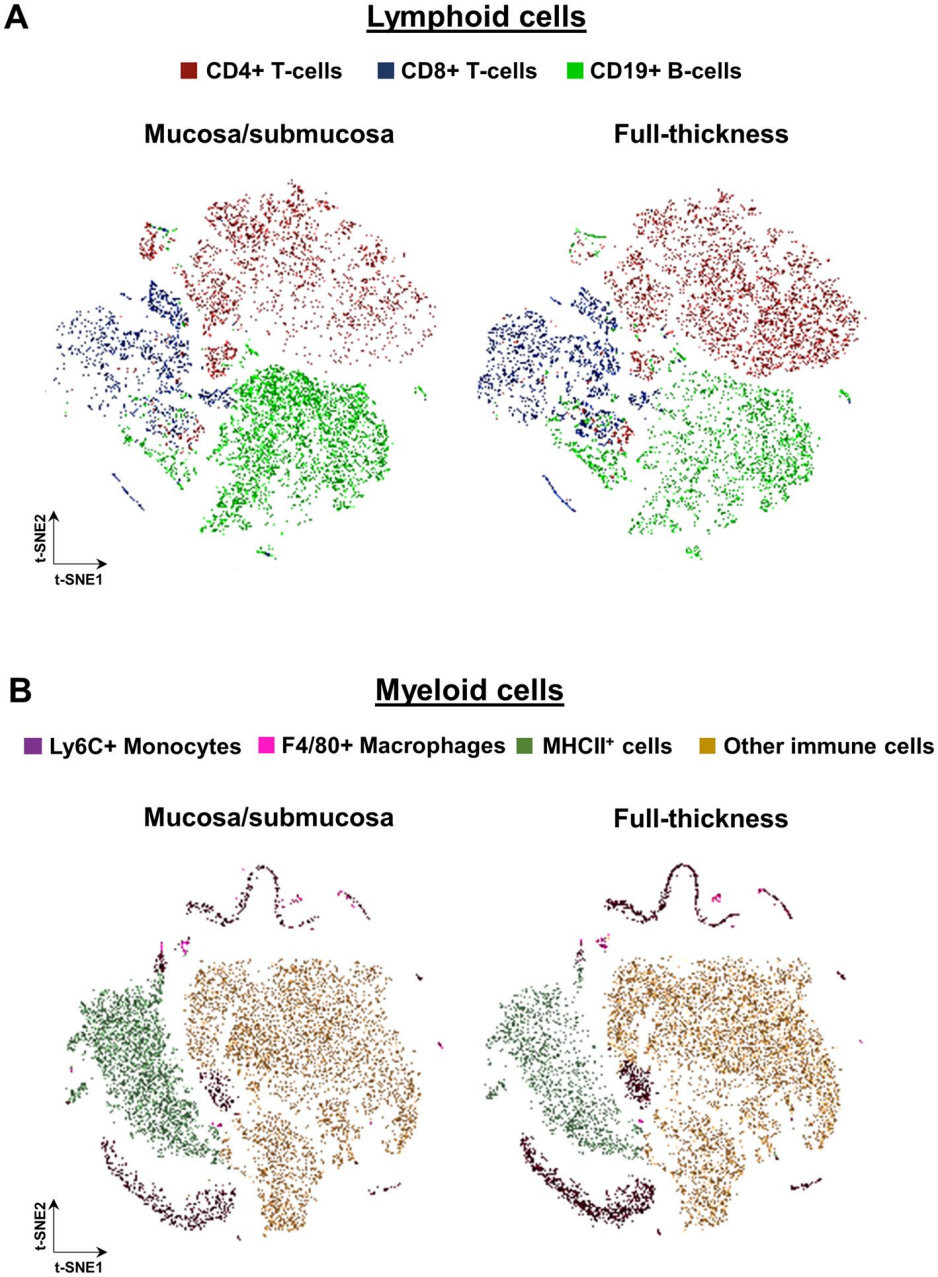
Experimental biases that are present when using mucosal/submucosal layers are eliminated in full-thickness samples of mouse colon. (**A**,**B**) t-SNE maps of the main populations of lymphoid (**A**) and myeloid (**B**) cells. CD19+ B-cells and MHCII+ cells are enriched in mucosal/submucosal samples at the expense of CD4+/CD8+ T-cells and LyC6+ monocytes, respectively.

To confirm that mechanical dissociation is well suited for recovering gastrointestinal immune cells from all colon layers at once, we then compared the relative abundance of the main types of lymphoid and myeloid cells in full-thickness *vs* mucosa/submucosa-only samples (all at P20, before weaning). Using t-SNE for global visualization in two dimensions, we notably found that mucosa/submucosa-only samples markedly overestimate the proportion CD19+ B-cells present in the colon (Fig. 5A) while the proportion of Ly6C+ monocytes is markedly underestimated (Fig. 5B). These experimental biases were found to be eliminated in full-thickness samples (Fig. 5), as also shown in representative contour plots (Fig.S1). These observations highlight the importance of including all colon layers for comprehensive characterization of the diverse populations of gastrointestinal immune cells.

### Development of antibody panels for global analysis of both innate and adaptative immune cells in the murine colon

Motivated by an interest for obtaining global snapshots of both innate and adaptative immune systems under various physiological contexts in the murine colon, we developed two antibody panels that target lymphoid and myeloid cell lineages separately. Selected markers were chosen based on existing literature^50,52,56,61–66^, also considering that we wanted our panels to be eventually useful in the context of inflammatory conditions as well. As detailed in Tables 1–2, these panels contain 26 antibodies in total (13 lymphoid-specific, 11 myeloid-specific, and 2 shared), mostly targeting extracellular markers (only two are targeting intracellular markers). Below is a summary of the flow cytometry gating strategies for each panel, using entire full-thickness colon samples mechanically dissociated from pre-weaned P20 mice as starting material.

**Figure 6:**
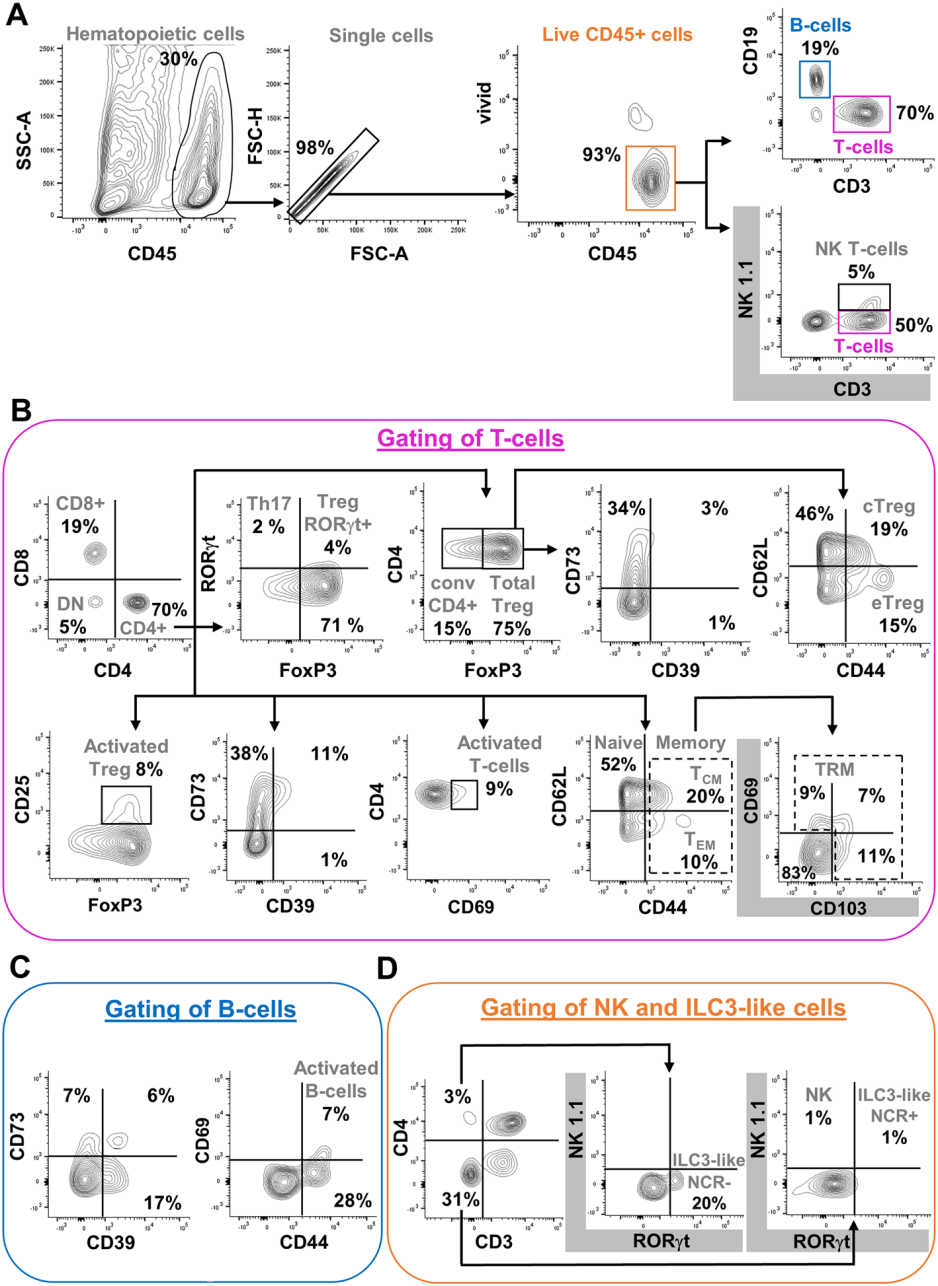
Gating strategy for the phenotypical characterization of lymphoid cell lineages from mechanically dissociated colon of P20 mice. (**A**) CD45+ hematopoietic cells are first gated for sequential exclusion of doublets/aggregated and dead cells. Single live CD45+ hematopoietic cells (orange box) are then gated to distinguish between B-cells (CD19+; blue box) and T-cells (CD3+; pink box). Alternatively, in a variant of the lymphoid panel (highlighted in grey), live CD45+ hematopoietic cells can first be gated to distinguish between natural killer T-cells (NK1.1+ CD3+; black box) and T-cells (NK1.1− CD3+; pink box). **(B)** As exemplified here for CD4+ T-cells (similar strategy for CD8+ T-cells and double-negative T-cells, except for T-helper 17), T-cells from the pink boxes in A can be further analyzed to evaluate the frequencies of regulatory (FoxP3+) and/or activated (CD25+ or CD69+) and/or effector (CD39+ and/or CD73+) and/or memory (CD44+ *vs* naïve CD62L+) subsets. ROR$\gamma$t further allows to identify T-helper 17 cells whereas, in the variant panel (highlighted in grey), CD103 further allows to identify tissue-resident memory cells (dashed boxes). (**C**) B-cells from the blue box in A can be further analyzed to evaluate the frequencies of effector (CD39+ and/or CD73+) and/or activated (CD69+) and/or memory (CD44+) subsets. **(D)** With the variant panel (highlighted in grey), non-T-cells (CD3− CD4−) from the orange box in A can be directly analyzed to distinguish between natural killer cells (NK1.1+) and innate lymphoid cells type 3 (ROR$\gamma$t+). NKT, natural killer T-cells; DN, double-negative T-cells; Th17, T-helper 17; conv, conventional; Treg, regulatory T-cells; cTreg, central Treg; eTreg, effector Treg; TRM, tissue-resident memory; NK, natural killer; ILC3, innate lymphoid cells type 3; NCR, natural cytotoxicity receptor.

With the lymphoid panel (Table 1 for antibodies list, Table 3 for gated phenotypes), single and live CD45+ cells can first be separated in B-cell and T-cell subsets (Fig. 6A; phenotypes in Table 3). From the T-cell subset, we can then evaluate CD4+ T-cells, CD8+ T-cells, and double-negative T-cells (Fig. 6B; phenotypes in Table 3). Of note, although subsequent steps shown are focused on the CD4+ T-cell subset, this is the same strategy for CD8+ and double-negative T-cell subsets (except for Th17 that is specific to the CD4+ T-cell subset). Hence, within CD4+, CD8+, and double-negative T-cells, frequencies of naïve, memory, and effector populations can be determined, along with activated cells and different populations of regulatory T-cells (Fig. 6B; phenotypes in Table 3). From the B-cell subset, we can also gate activated and effector cells (Fig. 6C; phenotypes in Table 3). Moreover, in a variant of the lymphoid panel, we replaced CD19 and CD25 markers by NK1.1 and CD103 markers (Table 1), thereby allowing to characterize natural killer cells, natural killer T-cells and ILC3-like populations, as well as to distinguish between tissue-resident and circulating memory cells (see graphs with grey axes in Fig. 6A,B,D; phenotypes in Table 3).

**Table 3:** Detailed phenotype of gated lymphoid cells in mice.

	Cell population	Markers	Main effector cytokines	References (for markers and/or cytokines)
Main populations	B-cells	CD3− CD19+	IL-2, IL-4, IL-6, IL-10, TGF-β	^62,96–98^
	CD4+ T-cells	CD3+ CD4+	IL-2, IL-4, IL-6, IL-10, IFN-$\gamma$	^62,97,99–101^
	CD8 T-cells	CD3+ CD8+	IL-2, IL-4, IL-6, IL-10, IL-22, IFN-$\gamma$	^62,97,100–103^
	double-negative T-cells	CD3+ CD4− CD8−	IL-2, IL-4, IL-10, IL-13, IL-17A, IFN-$\gamma$, TNF-α	^101,104–106^
B-cell subsets	CD73+	CD3− CD19+ CD73+	TBD The main function of B-cells is the production of antibodies	^62,107^
	CD39+	CD3− CD19+ CD39+		^108^
	activated	CD3− CD19+ CD44+ CD69+		^99^
CD4+ T-cell subsets	Th17	CD3+ CD4+ RORγt+	IL-17A, IL-17F, IL-21, IL-22	^62,103,109–111^
	naive	CD3+ CD4+ CD62L+ CD44−	none	^62,112–114^
	effector memory	CD3+ CD4+ CD62L− CD44+	IL-4, TNF-α, IFN-$\gamma$	^62,114–117^
	central memory	CD3+ CD4+ CD62L+ CD44+	IL-4, TNF-α, IFN-$\gamma$	^62,114,116,117^
	tissue-resident memory	CD3+ CD4+ CD44+ (CD103+ and/or CD69+)	IL-17, IFN-$\gamma$	^62,118–120^
	CD73+ and/or CD39+	CD3+ CD4+ (CD73+ and/or CD39+)	IFN-$\gamma ,$ TGF-β	^114,121,122^
	activated	CD3+ CD4+ CD69+	IL-21	^62,99,123^
	total Treg	CD3+ CD4+ FoxP3+	IL-10, IL-17, IFN-$\gamma$, TGF-β1	^4,62,97,109,111,113,124^
	activated Treg	CD3+ CD4+ FoxP3+ CD25+	IL-10, TGF-β	^62,114,125,126^
	CD73+ and/or CD39+ Treg	CD3+ CD4+ FoxP3+ (CD73+ and/or CD39+)	IL-10, TGF-β	^62,114,121,122,126,127^
	ROR $\gamma$ t+ Treg	CD3+ CD4+ FoxP3+ ROR $\gamma$ t+	IL-17	^128^
	effector memory Treg	CD3+ CD4+ FoxP3+ CD62L− CD44+	IL-10	^129,130^
	central memory Treg	CD3+ CD4+ FoxP3+ CD62L+ CD44+	IL-10	^129^
CD8+ T-cell subsets	naive	CD3+ CD8+ CD62L+ CD44−	none	^62,114^
	effector memory	CD3+ CD8+ CD62L− CD44+	IFN-$\gamma$	^62,102,114,116^
	central memory	CD3+ CD8+ CD62L+ CD44+	IFN-$\gamma$	^62,102,114,116^
	tissue-resident memory	CD3+ CD8+ CD44+ (CD103+ and/or CD69+)	IL-17A, IFN-$\gamma$	^120,131^
	CD73+ and/or CD39+	CD3+ CD8+ (CD73+ and/or CD39+)	IFN-$\gamma$	^132,133^
	activated	CD3+ CD8+ CD69+	IL-2, IFN-$\gamma$	^102,134^
	total Treg	CD3+ CD8+ FoxP3+	IL-2, IL-4, IL-10, IFN-$\gamma$	^4,97,100,125^
	activated Treg	CD3+ CD8+ FoxP3+ CD25+	IL-2	^132,135^
	CD73+ and/or CD39+ Treg	CD3+ CD8+ FoxP3+ (CD73+ and/or CD39+)	TBD	^127,132^
	ROR $\gamma$ t+ Treg	CD3+ CD8+ FoxP3+ ROR $\gamma$ t+	TBD	
	effector memory Treg	CD3+ CD8+ FoxP3+ CD62L− CD44+	IL-10	^125^
	central memory Treg	CD3+ CD8+ FoxP3+ CD62L+ CD44+	TBD	
Double-negative T-cell subsets	naive	CD3+ CD4− CD8− CD62L+ CD44−	none	
	effector memory	CD3+ CD4− CD8− CD62L− CD44+	TBD	^106^
	central memory	CD3+ CD4− CD8− CD62L+ CD44+	IFN-$\gamma$, TNF-α	^105^
	tissue-resident memory	CD3+ CD4− CD8− CD44+ (CD103+ and/or CD69+)	TBD	^105^
	CD73+ and/or CD39+	CD3+ CD4− CD8− (CD73+ and/or CD39+)	TBD	
	activated	CD3+ CD4− CD8− CD69+	IL-10	^101,105^
	total Treg	CD3+ CD4− CD8− FoxP3+	IL-10	^136,137^
	activated Treg	CD3+ CD4− CD8− FoxP3+ CD25+	TBD	^136,137^
	CD73+ and/or CD39+ Treg	CD3+ CD4− CD8− FoxP3+ (CD73+ and/or CD39+)	TBD	
	ROR $\gamma$ t+ Treg	CD3+ CD4− CD8− FoxP3+ ROR $\gamma$ t+	TBD	
	effector memory Treg	CD3+ CD4− CD8− FoxP3+ CD62L− CD44+	TBD	^137^
	central memory Treg	CD3+ CD4− CD8− FoxP3+ CD62L+ CD44+	TBD	^137^
	NKT cells	CD3+ CD4− CD8− NK1.1+	IL-21, IFN-$\gamma$, TNF-α	^62,97,113,123^
Innate lymphoid cells	NCR− ILC3− like	CD3− CD4+ NK1.1− ROR $\gamma$ t+	IL-17, IL-22, GM-CSF	^4,62,63,65,94,103,138,139^
	NCR+ ILC3− like	CD3− CD4− NK1.1+ ROR $\gamma$ t+	IL-22, IFN-$\gamma$, GM-CSF	^4,62,63,65,103,138^
	NK cells	CD3− CD4− NK1.1+ ROR$\gamma$t−	IL-12, IFN-$\gamma$	^62,97,101,140^

TBD, to be determined; Th17, T-helper 17; Treg, regulatory T-cells; NKT, natural killer T-cells; NCR, natural cytotoxicity receptor; NK, natural killer.

**Figure 7:**
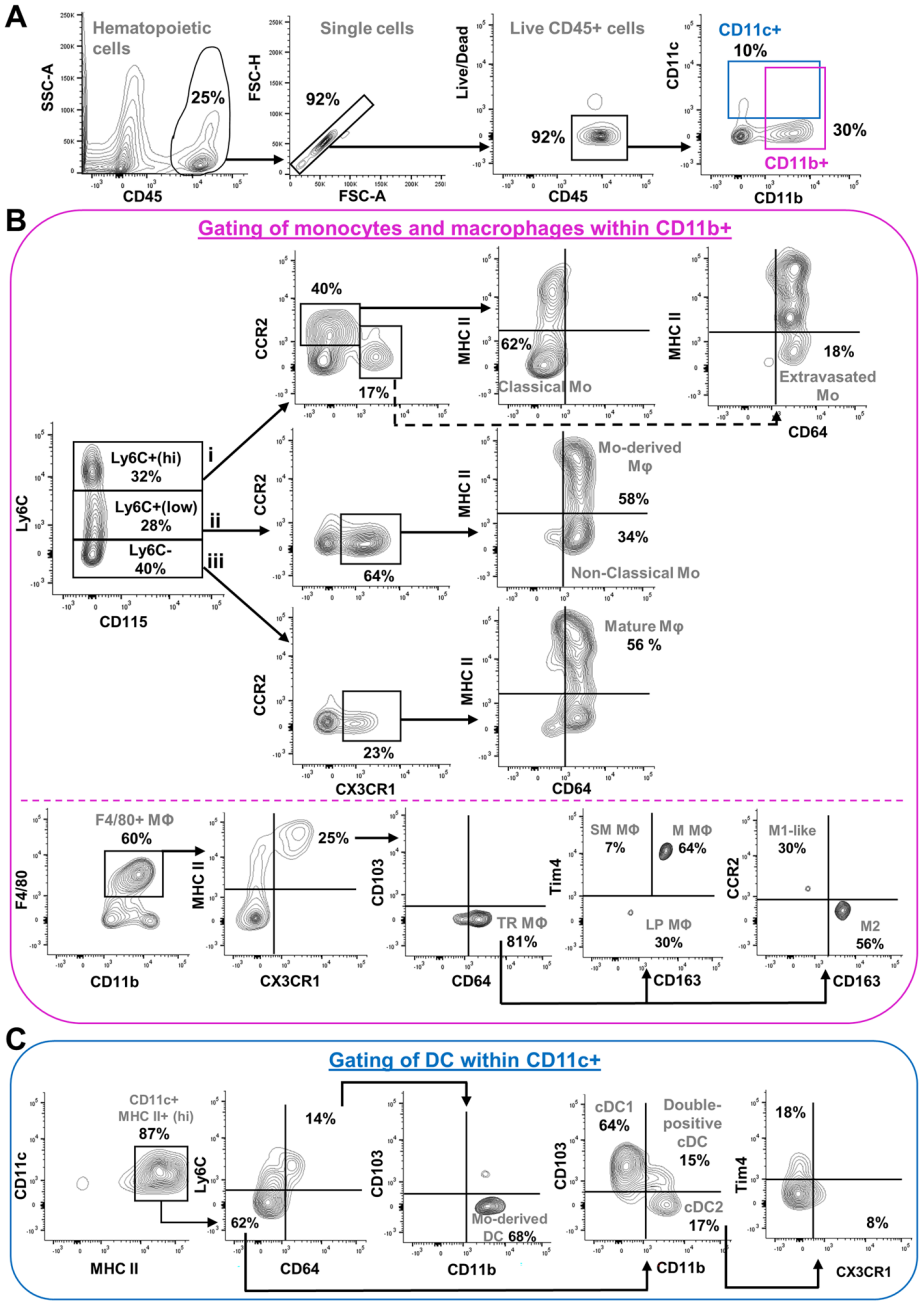
Gating strategy for the phenotypical characterization of myeloid cell lineages from mechanically dissociated colon of P20 mice. (**A**) CD45+ hematopoietic cells are first gated for sequential exclusion of doublets/aggregated and dead cells. Single live CD45+ hematopoietic cells are then gated to distinguish between CD11c+ (blue box) and CD11b+ (pink box) myeloid cells. (**B**) Panels above the dashed line show that CD11b+ cells from the pink box in A can be further analyzed to evaluate the frequencies of monocytes (LyC6+) and macrophages (LyC6-). Then, four additional markers (CCR2, CX3CR1, MHC II, CD64) allow to identify (i) classical and extravasated monocytes from the LyC6+ (hi) subset, (ii) non-classical monocytes and monocyte-derived macrophages from the LyC6+ (low) subset, and (iii) mature macrophages from the LyC6-negative subset. Panels below the dashed line show that CD11b+ F4/80+ macrophages can also be sequentially gated with MHCII/CX3CR1 and CD103/CD64 markers to evaluate the frequencies of tissue-resident macrophages, which can then be stratified for localization (lamina propria, submucosa, muscle) and inflammatory status (pro-inflammatory M1 *vs* anti-inflammatory M2) using three additional markers (CD163, Tim4 and CCR2). (**C**) Using four markers (Ly6C, CD64, CD103, CD11b), CD11c+ cells from the blue box in A can be further analyzed to distinguish between monocyte-derived dendritic cells and different subsets of conventional dendritic cells. Moreover, conventional dendritic cells type 2 can be further subdivided using Tim4/CX3CR1 markers. Mo, monocytes; MΦ, macrophages; TR, tissue-resident; LP, lamina propria; SM, submucosa; M, muscle; DC, dendritic cells; cDC, conventional DC.

With the myeloid panel (Table 2 for antibodies list, Table 4 for gated phenotypes), a first selection is made from single and live CD45+ cells using CD11b and CD11c antibodies (Fig. 7A). CD11b+ cells can then be analyzed to characterize monocytes and macrophages (Fig. 7B; phenotypes in Table 4), whereas CD11c+ cells can be analyzed to characterize dendritic cells (Fig. 7C; phenotypes in Table 4). From the LyC6+ monocyte subset, we can subsequently distinguish between classical monocytes, extravasated monocytes, non-classical monocytes, and monocyte-derived macrophages (Fig. 7B; phenotypes in Table 4). For F4 /80+ macrophages, we can further characterize tissue-resident macrophages, long-lived macrophages, sub-mucosal macrophages, muscular macrophages, lamina propria macrophages, M1-like macrophages, and M2 macrophages (Fig. 7B; phenotypes in Table 4). Finally, dendritic cell subsets can be analyzed to distinguish between monocyte-derived and conventional populations (Fig. 7C; phenotypes in Table 4). Here, it is important to bear in mind that myeloid cell populations share a lot of markers, leading to many possibilities in terms of gating strategies. What we described above is only one possible way to gate myeloid cells.

**Table 4:** Detailed phenotype of gated myeloid cells in mice.

	Cell population	Markers	Main effector cytokines	References (for markers and/or cytokines)
Monocyte subsets	Classical	CD11b+ Ly6C+ (hi) CCR2+ (hi) CX3CR1+ (low) MHCII− CD64−	IL-1β, IL-6, IL-12, IL-23, TNF-$\alpha$	^62,64,110,138,141–146^
	Extravasated	CD11b+ Ly6C+ (hi) CCR2+ (low) CX3CR1+ MHCII− CD64+	TBD	^141,142,144,146–148^
	Non-classical	CD11b+ Ly6C+ (low) CCR2− CX3CR1+ MHCII− CD64+	IL-10	^62,142–144,146^
Macrophage subsets	Mo-derived	CD11b+ Ly6C+ (low) CCR2+ (low) CX3CR1+ MHCII+ CD64+	IL-10	^141–144,146^
	mature	CD11b+ Ly6C− CCR2+ (low) CX3CR1+ MHCII+ CD64+	IL-10, IL-12,	^4,42,97,108,140,143,149^
	tissue-resident	CD11b+ F4/80+ CX3CR1+ MHC II+ CD64+ CD103−	IL-10, IL-23, IL-27	^33,42,62,138,141,144,145,150^
	sub-mucosal (long-lived)	CD11b+ F4/80+ CX3CR1+ MHCII+ CD64+ Tim4+ CD163−	IL-10, TNF-α, IFN-β	^151–153^
	muscularis (long-lived)	CD11b+ F4/80+ CX3CR1+ MHCII+ CD64+ Tim4+ CD163+	IL-10, TNF-α	^8,46,142,152,153^
	lamina propria	CD11b+ F4/80+ CX3CR1+ MHCII+ CD64+ Tim4−	IFN-β, IL-6, IL-10, IL-13, TNF-α, TGF-β	^42,142,151,152^
	M1 (pro-inflammatory)	CD11b+ F4/80+ CX3CR1+ MHCII+ CD64+ CCR2+ CD163−	IL-1β, IL-6, IL-12, IL-23, TNF-α , IFN-$\gamma$	^8,62,141–143,149,154,155^
	M2 (anti-inflammatory)	CD11b+ F4/80+ CX3CR1+ MHCII+ CD64+ CCR2− CD163+	IL-10, TGF-β	^8,46,62,141,142,149,154^
Dendritic cell subsets	Mo-derived	CD11c+ (hi) MHCII+ (hi) Ly6C+ CD64+ CD11b+ CD103−	TNF-α	^156^
	total conventional	CD11c+ (hi) MHCII+ (hi) Ly6C− CD64−	IL-1β, IL-2, IL-6, IL-7, IL-10, IL-12, IL-27	^62,97,98,118,143,150,155,157^
	conventional type 1	CD11c+ (hi) MHCII+ (hi) Ly6C− CD64− CD11b− CD103+	IL-4, IL-10, IL-12, IL-15, IL-27	^42,62,64,98,138,140,144–146,149,158,159^
	conventional type 2	CD11c+ (hi) MHCII+ (hi) Ly6C− CD64− CD11b+ CD103−	IL-6, IL-23	^42,62,64,138,144–146,158,159^
	double-positive conventional	CD11c+ (hi) MHCII+ (hi) Ly6C− CD64− CD11b+ CD103+	IL-6, IL-23	^64,98,144,145,155,158,159^
	CX3CR1+ conventional	CD11c+ (hi) MHCII+ (hi) Ly6C− CD64− CD11b+ CD103− CX3CR1+	IL-6, IL-23, IFN-β, TGF-β	^4,42,46,64,98,143–146^
	Tim4+ conventional	CD11c+ (hi) MHCII+ (hi) Ly6C− CD64− CD11b+ CD103− Tim4+	TBD	^153,160,161^

TBD, to be determined; Mo, monocytes.

In a final validation step, we sought to confirm that the very low detection of certain markers (NK1.1, CD39 in T-cells; CD115 in monocytes and macrophages) in the colon of P20 mice was biologically real and not due to an experimental artifact. To this end, we thus tested our panels on various samples from older weaned mice, including colon, whole blood and spleen. All markers that were weakly detected in the colon of P20 mice appeared markedly enriched in at least one of these additional sample types from P120 mice (Fig. S2). Therefore, we conclude that our antibody panels can efficiently provide global snapshots of both innate and adaptative immune systems, not only for colon samples but also for blood and spleen samples.

### Life stage-associated changes in the immune system profile of the proximal and distal colon from mice

**Figure 8:**
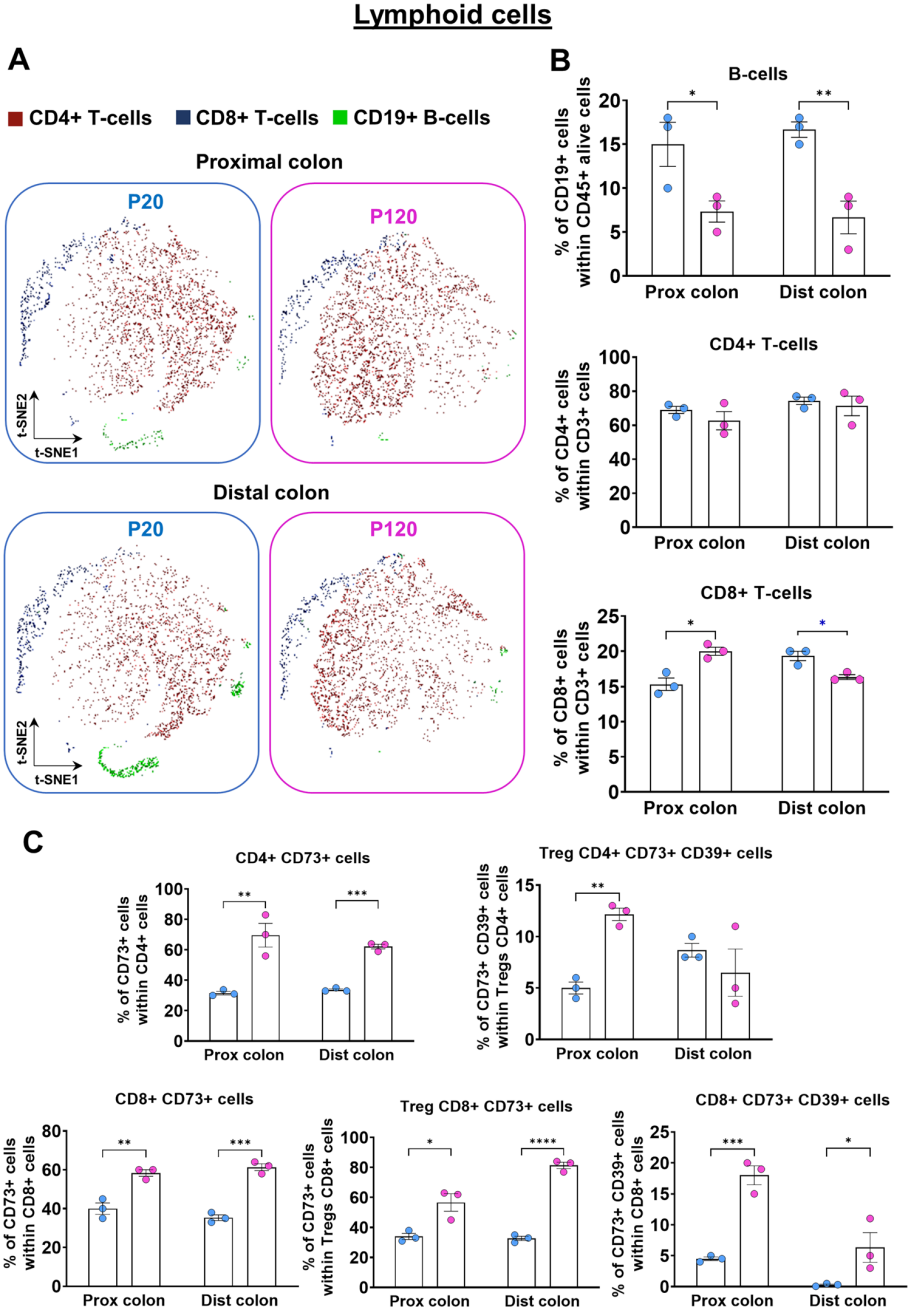
Life stage-associated changes in the lymphoid cell profile of the proximal and distal colon of FVB mice. (**A**,**B**) Representative t-SNE maps (**A**) and accompanying quantitative analyses (**B**) of the main populations of lymphoid cells show that total levels of CD19+ B-cells are lower at P120 than at P20, in both proximal and distal colon. Total levels of CD4+ T-cells are not affected by life stage, whereas total levels of CD8+ T-cells vary differently in proximal and distal colon. (**C**) Quantitative analyses of lymphoid cell subsets reveal specific life stage-related changes for several CD4+ and CD8+ subsets expressing CD73 and/or CD39. Treg, regulatory T-cells. N = 3 biological replicates; *P ≤ 0.05, **P ≤ 0.01, ***P ≤ 0.001 and ****P ≤ 0.0001; Student’s *t*-test.

**Figure 9:**
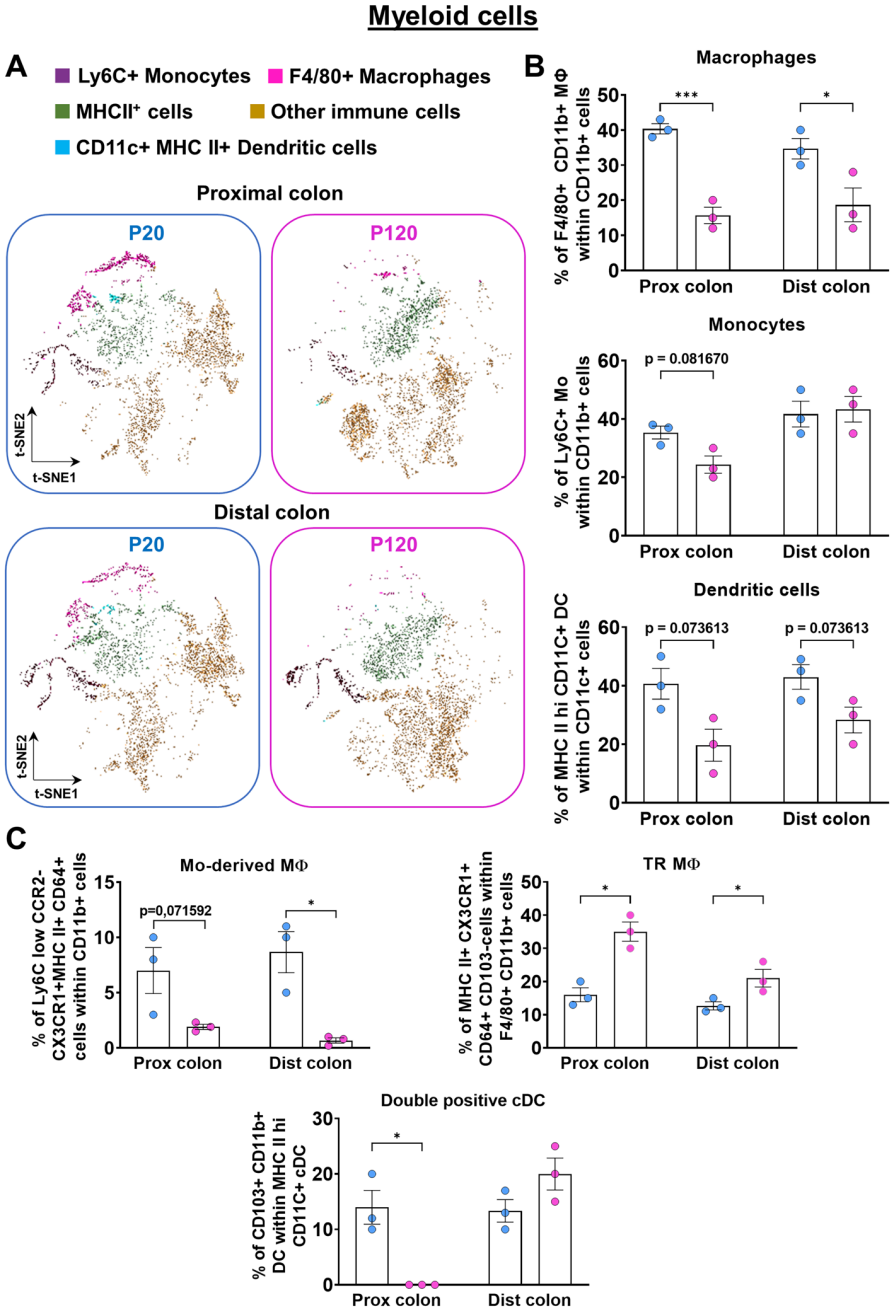
Life stage-associated changes in the myeloid cell profile of the proximal and distal colon of FVB mice. (**A**,**B**) Representative t-SNE maps (**A**) and accompanying quantitative analyses (**B**) of the main populations of myeloid cells show that total levels of CD11b+ F4/80+ macrophages are lower at P120 than at P20, in both proximal and distal colon. Total levels of LyC6+ monocytes are not significantly affected by life stage, whereas a general trend toward lower levels in adult mice is noted for MHC II+ CD11c+ dendritic cells. (**C**) Quantitative analyses of myeloid cell subsets reveal specific life stage-related changes for monocyte-derived macrophages (general decrease in adults) and tissue-resident macrophages (general increase in adults). Frequencies of CD11b+ CD103+ conventional dendritic cells are specifically lower in adult proximal colon, but not distal colon. Mo, monocytes; MΦ, macrophages; TR, tissue-resident; cDC, conventional dendritic cells. N = 3 biological replicates; *P ≤ 0.05 and ***P ≤ 0.001; Student’s *t*-test.

As a proof-of-concept of the usefulness of our approach, we conducted a systematic comparative analysis of two segments of the murine colon (full-thickness proximal *vs* full-thickness distal) as a function of age and weaning status corresponding to the adolescence-adulthood transition (pre-weaned P20 mice *vs* weaned P120 mice). With the lymphoid panel, we first evidenced that this transition impacts the frequencies of both CD19+ B-cells and CD8+ T-cells, but not overall levels of CD4+ T-cells (Fig. 8A,B). Interestingly, while the observed decline in CD19+ B-cells at P120 was found to be similar in both colon segments, the life stage-associated changes in CD8+ T-cells were instead found to be different in proximal (increased levels at P120) and distal (decreased levels at P120) segments (Fig. 8B). Upon more detailed analysis with the lymphoid panel, specific subsets of CD4+ T-cells were nonetheless found to be affected by the adolescence-adulthood transition as well. This is notably the case of CD4+ T-cell subsets also expressing the ectonucleotidases CD73 and CD39, which appeared generally increased at P120 as they are within the CD8+ T-cell population (Fig. 8C). With the myeloid panel, we detected a general decrease in the proportion of F4/80+ macrophages but not LyC6+ monocytes at P120 (Fig. 9A,B). A general adulthood-associated decrease in the frequency of CD11c+ MHC II+ dendritic cells was also noted, but without reaching statistical significance most likely because of small sample size (Fig. 9B). Upon more detailed analysis, an adulthood-associated decline in monocyte-derived macrophages (CD11b+ LyC6+ (low) CX3CR1+ CCR2+ (low) MHCII+ CD64+) was noted as well, reaching statistical significance in the distal colon only (Fig. 9C). In contrast, the frequency of tissue-resident macrophages (F4/80+ CD11b+ CX3CR1+ MHC II+ CD64+ CD103−) was found to significantly increase during the adolescence-adulthood transition in both the proximal and distal colon (Fig. 9C). Finally, a major decrease in the frequency of a specific subset of conventional dendritic cells (CD11c+ MHC II+ Ly6C− CD64− CD11b+ CD103+) was noted at P120, but only in the proximal colon. Altogether, these findings thus confirm that our combination of mechanical dissociation with both antibody panels described herein is useful for detecting physiologically relevant changes in the composition of the gastrointestinal immune system in mice.

## Discussion

The main goal of the current study was to identify a relatively simple method for global profiling of lymphoid and myeloid cell lineages from full-thickness mouse colon samples. Comparison of enzymatic and mechanical cell dissociation techniques via flow cytometry showed that the mechanical approach best balances total cell recovery yields with cell viability (Fig. 2) and presence of cell-surface markers in lymphoid and myeloid cells. Both tested enzyme-based techniques were found to be globally deleterious for the analysis of lymphoid lineages, presumably because of proteolytic cleavage of the cell surface markers of interest. Although the Collagenase V & Liberase LT cocktail performed better than Collagenase I & Dispase II for detecting CD19+ B-cells, both cocktails were found to be similarly much less efficient than the mechanical technique for detecting CD3+ T-cells (Fig. 3A,B). Both cocktails were also found to be similarly less efficient for detecting CD8+ T-cells, with the Collagenase I & Dispase II cocktail being in addition particularly inefficient for detecting CD4+ T-cells as well (Fig. 3C,D). Such negative impact of enzymatic cell dissociation techniques on lymphoid lineages has been previously documented in several studies^38,51,52,67–69^. This can have profound consequences when comes time to draw biologically valid conclusions. For example, based on our CD4/CD8 profiling data, both enzymatic techniques would have led to grossly erroneous conclusions about the real frequency of double negative T-cells (Fig. 3C,D). Intriguingly, enzymatic dissociation appears to be less problematic for myeloid cells. Notably, we found that both tested enzymatic cocktail were either equally efficient or even slightly more efficient than mechanical dissociation for detecting CD11b+ F4/80+ macrophages (Fig. 4A,B). However, this was not the case for CD11c+ MHCII+ dendritic cells and CD11b+ LyC6+ monocytes, with the latter being even again more represented using the mechanical approach (Fig. 4C–F). In the end, we thus think that the far superior performance of the mechanical technique with lymphoid cells in general (and monocytes) greatly outweighs the slight advantage of enzymatic-based approaches in detecting macrophages. Yet, we do recognize that mastering the mechanical technique with full-thickness mouse colon samples requires a fair amount of practice for finding the right level of manual strength to use (Movie S2). Moreover, the needed level of manual strength to use will likely be different in other segments of the gastrointestinal tract (e.g., small intestine), which is important to consider if one wants to combine multiple segments in the same analysis. When tissue crushing is too harsh (Movie S3), this leads to low cell yields and high mortality rates of immune cells, as previously reported in some studies^38,70^. Nonetheless, other studies have shown that even when mortality rates are high, mechanically dissociated immune cells that survive remain more immunocompetent than those obtained following collagenase digestion^71^.

Another key goal of this study was to develop robust antibody panels that would allow to profile a wide array of lymphoid and myeloid cells in a cost-effective manner. These panels were designed to be used on a widely used multi-color flow cytometer with minimal configuration, thereby facilitating their use by the research community. For research teams having access to a multi-color flow cytometer with cell sorting capacity, Tables S1 and S2 show that several of the cell populations that can be identified with our panels and gating strategies can also be recovered in sufficient amounts for further downstream analyses. As proof-of-concept of the usefulness of our panels for detecting physiologically relevant changes in the gastrointestinal immune system, we analyzed the impact of life stages (pre-weaned juvenile *vs* weaned adult mice) and anatomical region (proximal *vs* distal colon) on immune cell profiles. Both age^32,72,73^ and weaning status (via food and/or microbiome-related changes)^74–76^ are well-known determinants of immune system composition and function in the gastrointestinal tract, as we observed with both of our panels (Figs. 8–9). What was a bit more surprising was to find that some of these changes were completely different at each end of the colon, such as the adulthood-associated change in the frequency of CD8+ T-cells which is increased in proximal colon but decreased in distal colon (Fig. 8B). Differences in immune system composition and function are expected when comparing major anatomical subdivisions of the gastrointestinal tract, like the colon and the small intestine^77,78^. Our data now indicate that such differences can also be present in juxtaposed segments of the same major subdivision of the gastrointestinal tract. This clearly adds to the notion that care should be taken not to systematically extrapolate findings made in one region of the gastrointestinal tract to another region.

Our focus on pre-weaned P20 mice is due to our general interest for Hirschsprung disease, as most mouse models for this life-threatening condition normally succumb soon after weaning (from P21 onwards)^79–81^. As seen in human patients^82,83^, these mice develop Hirschsprung-associated enterocolitis (HAEC) which is then believed to progress to sepsis and thus to be the main cause of death for Hirschsprung disease^84^. However, how exactly HAEC develops is not well understood. Like other inflammatory conditions affecting the gastrointestinal tract, such as Crohn’s disease, an immune system aspect is presumably central, but this question has not been addressed in a global manner yet. Immune cell infiltration in mouse models of Hirschsprung disease is frequently reported using histology techniques only^60,85,86^, without further detailed analysis. Otherwise, the very few studies that have investigated this question in a more detailed manner were mostly restricted to Peyer’s patches^87,88^, based on the prior discovery that the Hirschsprung disease-associated signaling pathway GDNF-RET is key for the development of this lymphoid site in the ileum^89,90^. Notably, smaller Peyer’s patches in *Ednrb*-mutant mice have been associated with a depletion of both B-cells and immunoglobulins^87,88^. Another study with different mouse models of defective EDN3-EDNRB signaling reported more generalized lymphoid depletion, also impacting CD4+ /CD8+ T-cells and involving other lymphoid organs (bone marrow, spleen and thymus)^91^. A more recent study, again performed with an *Ednrb*-mutant mouse model, strongly suggests that macrophage activation in the colon locally contributes to the etiology of HAEC as well^92^. All these prior observations highlight the need for more comprehensive analyses of the immune system at the site of HAEC development (i.e., in the colon instead of the small intestine), ideally using various genetically distinct mouse models of Hirschsprung disease—a complex genetic disease^93^. Performing that kind of analysis using the method described herein will be especially important in the context of GDNF-based treatment of Hirschsprung disease^58,60^, as rectally administered GDNF is expected to not only locally stimulate neurogenesis but also directly influence numerous immune cell populations^94,95^. Such studies are currently being completed in our laboratories and will be the subject of a forthcoming publication. That said, our results already allow us to attest that our general approach works equally well with inflamed tissue, and that it can reliably detect both disease-associated and treatment-induced changes.

In conclusion, we expect that the experimentally validated approach described herein will be useful for a large number of laboratories interested in globally assessing the immune status of the mouse bowel, in a wide variety of experimental contexts. This contribution is thus an effort to democratize such analyses, including laboratories with little or no previous experience in the field.

### Supplementary Information

Supplementary Video 1.

Supplementary Video 2.

Supplementary Video 3.

Supplementary Information 1.

## Data Availability

All relevant data are within the manuscript and its Supplementary Information files.
