# Supplementary material for: Efficient enzyme-free method to assess the development and maturation of the innate and adaptive immune systems in the mouse colon
Source: Sci Rep. 2024 May 14;14:11063. doi: 10.1038/s41598-024-61834-5 (PMC11094196; doi:10.1038/s41598-024-61834-5)
Supplement: Supplementary file 4 — Supplementary Information 1. [file 41598_2024_61834_MOESM4_ESM.docx]

**Supplementary Information**

**Figure S1. Representative contour plots of B-cells, T-cells and monocytes in mucosa and full-thickness colon of juvenile mice.**

**Figure S2.** **Validation of markers that are hardly detected in the colon of juvenile mice.**

**Table S1. Average number of gated lymphoid cell subtypes following mechanical dissociation of the entire full-thickness colon (~6cm in length) from P20 FVB mice.**

**Table S2. Average number of gated myeloid cell subtypes following mechanical dissociation of the entire full-thickness colon (~6cm in length) from P20 FVB mice.**

**Movie S1. Manual crushing of mouse spleen.**

**Movie S2. Adequate manual crushing of mouse colon.**

**Movie S3. Inadequate manual crushing of mouse colon.**

**
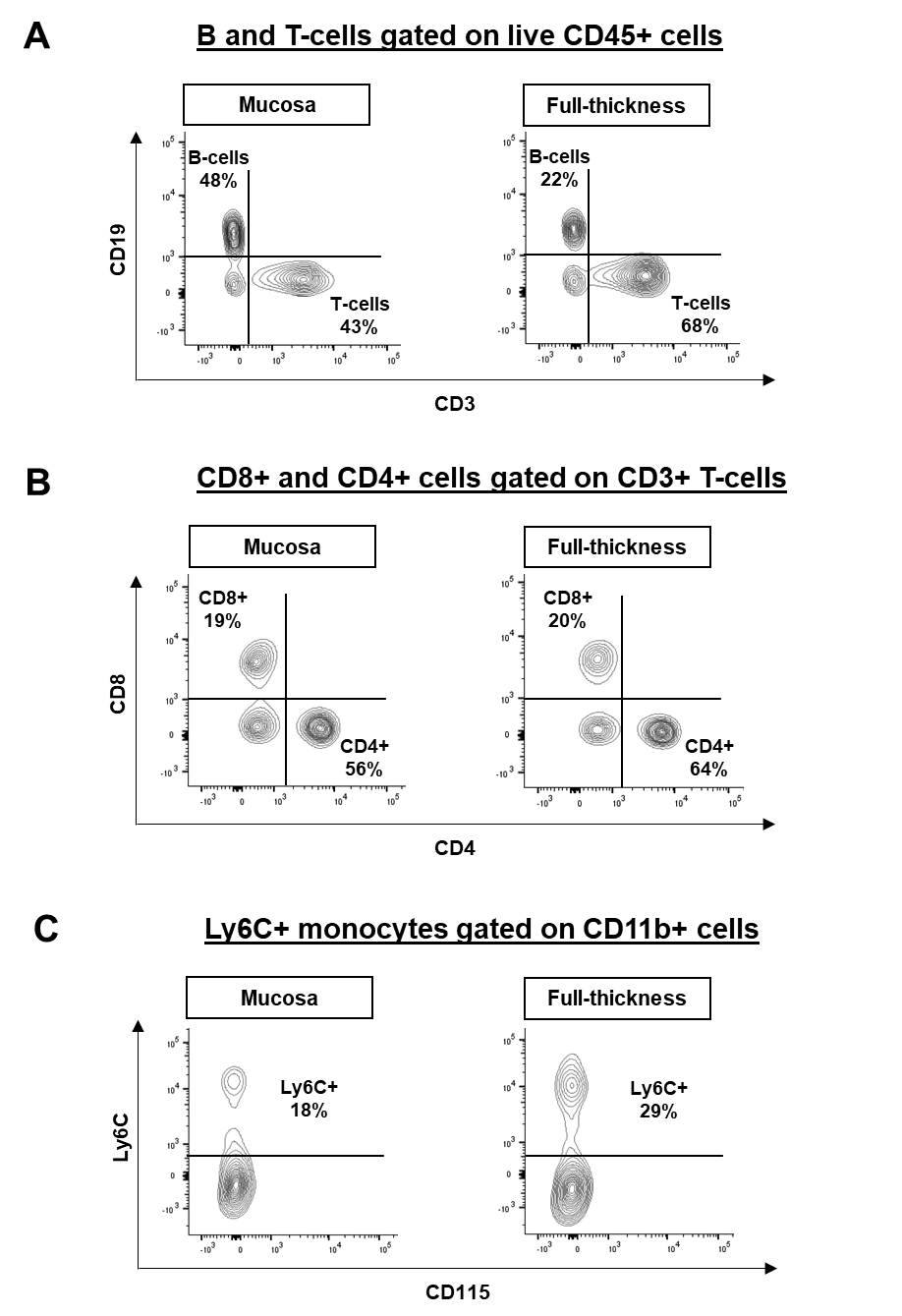
Figure S1. Representative contour plots of B-cells, T-cells and monocytes in mucosa/submucosa and full-thickness colon of juvenile mice. (A-B)** CD19+ B-cells and CD3+ T-cells. **(B)** CD4+ and CD8+ T-cells. **(C)** Ly6C+ CD11b+ monocytes.

**
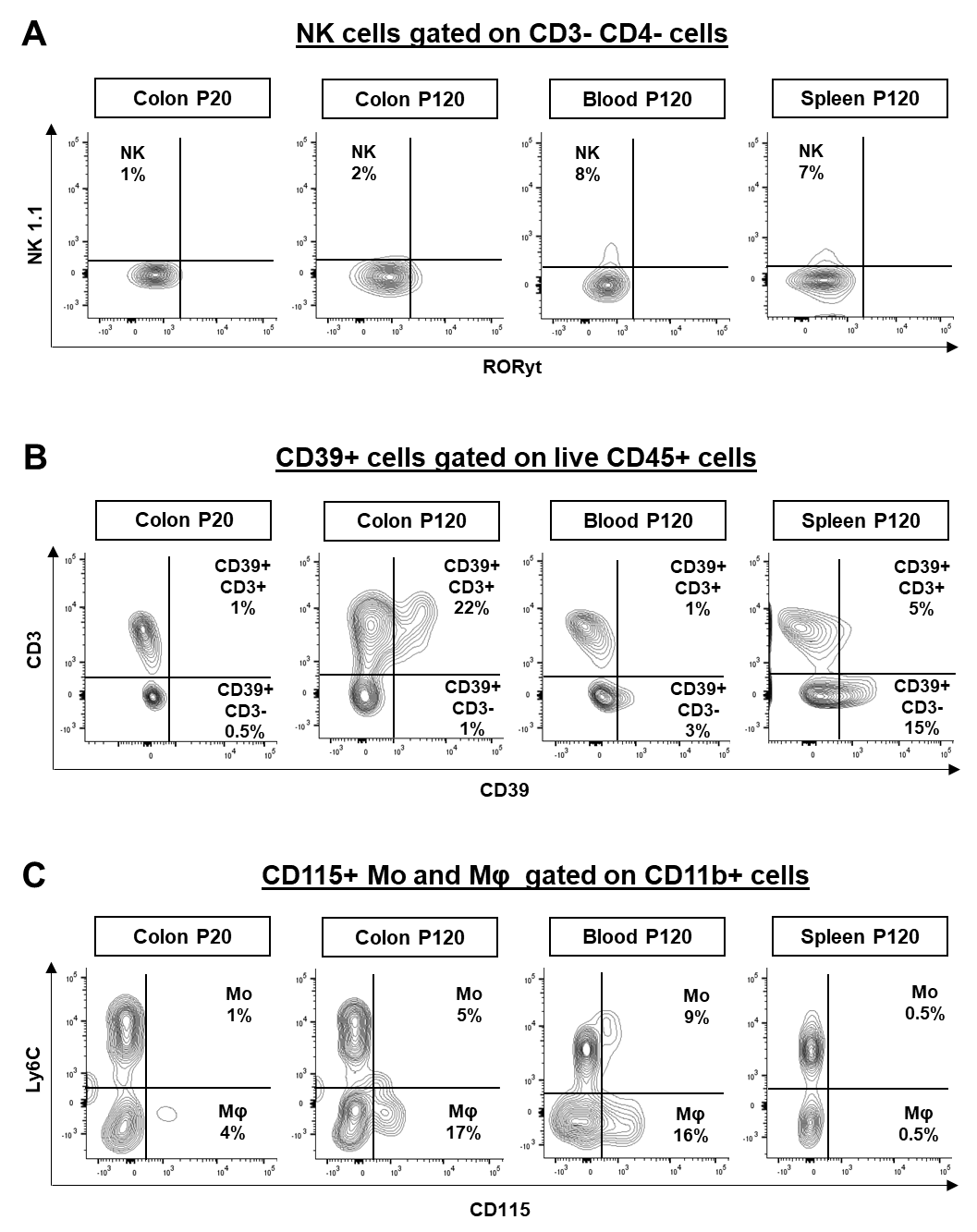
**

**Figure S2.** **Validation of markers that are hardly detected in the colon of juvenile mice. (A)** Representative contour plots of NK1.1 expression by natural killer cells in the colon at P20 and in colon, blood and spleen at P120. **(B)** Representative contour plots of CD39 expression by CD3+ T-cells in the colon at P20 and in colon, blood and spleen at P120. **(C)** Representative contour plots of CD115 expression by monocytes and macrophages in the colon at P20 and in colon, blood and spleen at P120. NK, natural killer; Mo, monocytes; MΦ, macrophages.

**Table S1. Average number of gated lymphoid cell subtypes following mechanical dissociation of the entire full-thickness colon (~6cm in length) from P20 FVB mice.**

|  | Cell population | Cell # |
| --- | --- | --- |
| Main populations | B-cells | 6 009 |
|  | CD4+ T-cells | 12 800 |
|  | CD8+ T-cells | 3 872 |
|  | double-negative T-cells | 710 |
| B-cell subsets | CD73+ | 1 464 |
|  | CD39+ | 1 650 |
|  | activated | 1 105 |
| CD4+ T-cell subsets | Th17 | 763 |
|  | naive | 6 141 |
|  | effector memory | 1 570 |
|  | central memory | 5 035 |
|  | tissue-resident memory CD103+ | 1 568 |
|  | tissue-resident memory CD69+ | 220 |
|  | tissue-resident memory CD103+ CD69+ | 247 |
|  | CD73+ | 4 490 |
|  | CD39+ | <50 |
|  | CD73+ CD39+ | 105 |
|  | activated | 866 |
|  | total Treg | 1 460 |
|  | activated Treg | 1 007 |
|  | CD73+ Treg | 576 |
|  | CD39+ Treg | <50 |
|  | CD73+ CD39+ Treg | 109 |
|  | RORγt+Treg | 85 |
|  | effector memory Treg | 183 |
|  | central memory Treg | 445 |
| CD8+ T-cell subsets | naive | 2 127 |
|  | effector memory | 502 |
|  | central memory | 1 082 |
|  | tissue-resident memory CD103+ | 111 |
|  | tissue-resident memory CD69+ | <50 |
|  | tissue-resident memory CD103+ CD69+ | <50 |
|  | CD73+ | 1 430 |
|  | CD39+ | <50 |
|  | CD73+ CD39+ | 108 |
|  | activated | 170 |
|  | total Treg | 236 |
|  | activated Treg | 162 |
|  | CD73+ Treg | 88 |
|  | CD39+ Treg | <50 |
|  | CD73+ CD39+ Treg | <50 |
|  | RORγt+Treg | <50 |
|  | effector memory Treg | <50 |
|  | central memory Treg | 61 |
| Double-negative T-cell subsets | naive | 81 |
|  | effector memory | 457 |
|  | central memory | 138 |
|  | tissue-resident memory CD103+ | 143 |
|  | tissue-resident memory CD69+ | <50 |
|  | tissue-resident memory CD103+ CD69+ | <50 |
|  | CD73+ | 606 |
|  | CD39+ | <50 |
|  | CD73+ CD39+ | <50 |
|  | activated | 255 |
|  | total Treg | 189 |
|  | activated Treg | 102 |
|  | CD73+ Treg | 140 |
|  | CD39+ Treg | <50 |
|  | CD73+ CD39+ Treg | <50 |
|  | RORγt+ Treg | <50 |
|  | effector memory Treg | 127 |
|  | central memory Treg | <50 |
|  | NKT cells | 186 |
| Innate lymphoid cells | NCR- ILC3-like | 74 |
|  | NCR+ ILC3-like | <50 |
|  | NK cells | 125 |

**Table S2. Average number of gated myeloid cell subtypes following mechanical dissociation of the entire full-thickness colon (~6cm in length) from P20 FVB mice.**

|  | Cell population | | Cell # |
| --- | --- | --- | --- |
| Main populations | Monocytes | 2 015 | |
|  | Macrophages | 7 109 | |
|  | Dendritic cells | 1 160 | |
| Monocyte subsets | Classical | | 760 |
|  | Extravasated | | 81 |
|  | Non-classical | | 483 |
| Macrophage subsets | Mo-derived | | 550 |
|  | mature | | 2 531 |
|  | tissue-resident | | 1 096 |
|  | sub-mucosal (long-lived) | | 114 |
|  | muscularis (long-lived) | | 296 |
|  | lamina propria | | 670 |
|  | M1 (pro-inflammatory) | | 210 |
|  | M2 (anti-inflammatory) | | 362 |
| Dendritic cell subsets | Mo-derived | | 120 |
|  | conventional type 1 | | 536 |
|  | conventional type 2 | | 294 |
|  | double-positive conventional | | 168 |
|  | CX3CR1+ conventional | | <50 |
|  | Tim4+ conventional | | <50 |
